# Chronic stress and functional health in older adults with concerns about falling: a study protocol of a randomized controlled trial with multicomponent exercise intervention (FEARFALL)

**DOI:** 10.1186/s13063-024-08462-6

**Published:** 2024-09-20

**Authors:** Sabine Britting, Robert Kob, Anja Görlitz, Cornel C. Sieber, Ellen Freiberger, Nicolas Rohleder

**Affiliations:** 1 Institute for Biomedicine of Aging, Friedrich-Alexander-Universität Erlangen-Nürnberg, Kobergerstraße 60, Nuremberg, Bavaria, 90408 Germany; 2 Department of Psychology, Chair of Health Psychology, Friedrich-Alexander-Universität Erlangen-Nürnberg, Erlangen, Bavaria Germany; 3 Department of Medicine, Kantonsspital Winterthur, Winterthur, Switzerland

**Keywords:** Older adults, Concerns about falling, Inflammation, Sarcopenia, Randomized controlled trial, Chronic stress, Perceived stress, Interleukin 6, Cortisol

## Abstract

**Background:**

Maintenance of physical function, mobility, and independent living are important goals for older adults. However, concerns about falling (CaF) play a central role in the vicious cycle of CaF, inflammation, loss of muscle mass, and decreasing physical function ultimately resulting in negative health outcomes. CaF, like other states of chronic stress and anxiety, can be considered as enduring adverse stimuli affecting the stress systems and the inflammatory system. Therefore, the aim of this study is to investigate whether a reduction of CaF leads to a reduction of stress and therefore possibly reduces chronic low-grade inflammation. Understanding the role and directionality of the effects of inflammation on CaF increases our understanding of age-related loss of mobility and physical function.

**Methods:**

In this study, community-dwelling older adults, aged 70 years and older, will be randomly assigned to either a 4-month, multi-component intervention with exercise training and cognitive-behavioral components or to a sham control group with light stretching exercises, cognitive training, and educational health lectures. For the operationalization of specific CaF, the Falls Efficacy Scale—International will be used. Stress and related psychological symptoms will be monitored using established self-reports and by measuring salivary cortisol. Concentrations of C-reactive protein, interleukin 6, interleukin 10, and tumor-necrosis-factor-alpha, as well as gene expression of selected inflammatory transcripts, will be used as surrogate parameters of the inflammatory status at baseline, after the 4-month intervention and 8-month follow-up.

**Discussion:**

This study will be the first to test whether CaF are related with stress system activity or reactivity or with markers of inflammation in the context of a multi-component intervention with exercise training and cognitive-behavioral components addressing CaF. The reduction of specific CaF or general psychological symptoms should reverse alterations in stress systems, and / or slow down low-grade inflammation. Changes in activity, as well as psychological and biological pathways leading from CaF to muscle loss will be measured, to disentangle the individual contribution to sarcopenia, and to provide an additional pathway to break or slow-down the vicious cycle of CaF and sarcopenia.

**Trial registration:**

German Clinical Trials Register (DRKS): DRKS00029171. Registered 22 July 2022.

**Supplementary Information:**

The online version contains supplementary material available at 10.1186/s13063-024-08462-6.

## Administrative information

Note: the numbers in curly brackets in this protocol refer to SPIRIT checklist item numbers. The order of the items has been modified to group similar items (see http://www.equator-network.org/reporting-guidelines/spirit-2013-statement-defining-standard-protocol-items-for-clinical-trials/).

**Table:** 

Title {1}	Chronic Stress and functional Health in older adults with concerns about falling: A study protocol of a randomized controlled trial with multicomponent exercise intervention (FEARFALL).
Trial registration {2a and 2b}.	German Clinical Trials Register (DRKS): DRKS00029171. Registered 22 July 2022, https://drks.de/search/de/trial/DRKS00029171.
Protocol version {3}	Version 1.0 dated on 13 March 2024
Funding {4}	FEARFALL is funded by the Deutsche Forschungsgemeinschaft (DFG, German Research Foundation) – 465677295
Author details {5a}	Sabine Britting, Robert Kob, Anja Görlitz, Cornel Sieber, Ellen Freiberger, Nicolas Rohleder
Name and contact information for the trial sponsor {5b}	The FAU is the sponsor of this trial (investigator-initiated trial). SB and NR are the principal investigators of FEARFALL. Contact information: Friedrich-Alexander-Universität Erlangen-Nürnberg, Institute of Biomedicine of Aging, Kobergerstraße 60, 90,408 Nuremberg, sabine.britting@fau.de; Friedrich-Alexander-Universität Erlangen-Nürnberg, Chair of Health Psychology, Nägelsbachstr. 49a, 91052 Erlangen; nicolas.rohleder@fau.de.
Role of sponsor {5c}	FEARFALL is an investigator-initiated trial. Therefore, the sponsor is responsible for planning, conducting, analysis and exploitation of the study.

## Introduction

### Background and rationale {6a}

The increasing life expectancy among older populations presents unique challenges in today’s western societies [1]. Maintenance of physical function, mobility, and the possibility to live independently are important for older adults with regard to health and quality of life. However, this goal is counteracted by the age-related loss of physical function as well as a decreased level of strength and muscle mass. Furthermore, the degenerative process could be reinforced if the older person avoids physical activity and exercise, e.g., due to concerns about falling (CaF). The prevalence of CaF was reported between 20 and 85% in older adults. About 10% of community-dwelling older adults experience excessive CaF [2]. CaF has been identified as an independent risk factor for reduced quality of life, increasing fall risks [3–6], and endangering the independence of community-dwelling older adults. The main consequence is a restriction of activities leading to a downward spiral of inactivity, deconditioning, and loss of confidence, thereby contributing to the development of sarcopenia [7–11].

In 2018, Cruz-Jentoft et al. published a new operational definition of sarcopenia in the name of the “European Working Group on Sarcopenia in Older persons” (EWSGOP2) [12]. The operational definition of sarcopenia includes (1) low muscle strength, (2) low muscle quality or quantity, and (3) low physical performance. Sarcopenia is a progressive and generalized skeletal muscle disorder increasing the risk of adverse outcomes including falls, fractures, physical disability, and mortality. Due to the increasing risk of hospitalization, and increased costs during hospital care, sarcopenia also represents an economic burden for health care systems [13]. Prevalence of sarcopenia varies widely due to different cut-off scores and is reported “with regional and age-related variations between, 1–29% in community-dwelling populations, 14–33% in long-term care populations, and 10% in the only acute hospital-care population examined” [14].

Lopez et al. published a recent article on “the Hallmarks of Aging” where the underlying mechanisms as the biochemical and molecular processes that cause aging address twelve mechanisms. One of these is chronic inflammation, which increases with aging (“inflammaging”). Inflammaging is accompanied by elevated levels of circulating inflammatory cytokines and biomarkers, e.g., C-reactive protein (CRP) and interleukin 6 (IL-6). For all-cause mortality in aging human population, elevated IL-6 concentrations serve as predictive biomarkers [15]. Inflammaging may result from multiple causes such as the accumulation of pro-inflammatory tissue damage, which play a role in obesity and type 2 diabetes [16]. Recent evidence has related acute and chronic stress to the inflammatory pathways in the aging process.

A substantial body of research showed that acute, as well as chronic stress, profoundly affects stress systems, including the hypothalamic–pituitary–adrenal (HPA) axis and the sympathetic nervous system (SNS) [17]. Acute stress induces transient increases in stress system activity and widespread effects on target tissues throughout the entire organism [18]. Long-term exposure to adverse psychological conditions, including chronic stress and anxiety, alters basal activity of stress systems [19] and, more importantly, leads to alterations with pathophysiological importance in cells and tissues throughout the organism. Of particular importance in chronic stress is inflammation. Longitudinal studies, for example in family caregivers of chronically ill relatives, have shown that chronic stress is associated with increases in peripheral inflammatory activity [20]. Stress-induced increases in systemic, low-grade inflammation are a strong candidate for linking chronic stress with disease, because the role of inflammation in predicting morbidity and mortality is undisputed [21]. Inflammation has been identified as a key pathophysiological process in cardiovascular disease, cancer, diabetes, and—most importantly—in age-related diseases such as frailty and sarcopenia [22].

As stated above, CaF has demonstrated an impact for development, maintenance, and exacerbation of sarcopenia [23–25]. One barrier in this field are the different constructs and terms related to CaF [26, 27]. At present, there are different terms that refer to the psychological effects of falls, including, e.g., “concerns about falling”, “fear of falling”, “fall-related anxiety”, or “balance confidence” and “falls-efficacy”. Underlying these concepts are related—but distinct—psychological constructs, which have led to considerable complexity and confusion within the literature. The term “fear of falling” is frequently used in the scientific literature. Nevertheless, the recent “World Falls Guidelines” recommend that clinicians use the term “concerns about falling” (CaF), primarily because the older adult panel preferred the term “concern” over “fear” [27].

In a recent qualitative study, Ellmers and colleagues demonstrated that CaF develop in response to one’s perception of their “aging body” and recognition of their vulnerability to suffering severe injuries, rather than the fall itself. Accordingly, individuals with high CaF tend to be primarily concerned about the anticipated long-term consequences of an injurious fall (e.g., loss of independence) [28]. In addition, CaF demonstrated an impact on gait as older adults with high CaF can adopt a more “cautious gait” by reduced velocity, taking shorter steps, widening the base of support, and spending more time in double limb support [29].

In a preliminary study, we found that these CaF are not only leading to decreased physical activity but are also associated with elevated levels of peripheral inflammation (unpublished results). In this analysis, plasma IL-6 was positively related with self-reported CaF, in older women with sarcopenic obesity, who had experienced a fall event in the past. Accordingly, long-term exposure to adverse psychological conditions, including chronic stress and anxiety, has previously been associated to the upregulation of peripheral inflammatory activity. This is consistent with previous findings that chronic low-grade activation of the immune system has been shown to intensify the decay of skeletal muscle [30–32]. Therefore, a vicious, feed-forward cycle of CaF, inflammation, loss of muscle mass, and decreasing physical function is created that ultimately results in negative health outcomes, i.e., falls, dependence, and death. Additionally, exercise training alone might not disturb this cycle because a person with elevated CaF would exhibit an increasing stress level and thus more inflammatory cytokines blunting the anabolic effects of the intervention. Therefore, this study will utilize a multi-component intervention with exercise training and cognitive-behavioral components to oppose muscular decay as well as address CaF in older adults.

To the best of our knowledge, no published study to date has directly tested whether CaF are related with stress system activity or with any marker of inflammation. Understanding the role and directionality of the effects of inflammation on CaF would significantly increase our understanding of the underlying causes of age-related loss of mobility and open up a number of avenues that would increase the efficacy of interventions aimed at increasing mobility and functional health in later life.

### Objectives {7}

The main and overarching objective of the project is to understand the role of the psychobiological stress response, including stress system alterations and inflammation, in the vicious cycle between CaF, declines in mobility, and development of muscle loss and sarcopenia. Therefore, a well-established intervention aimed at reducing CaF and increasing activity will be performed, to test whether a reduction of CaF can break the vicious cycle, and stop the downward spiral towards sarcopenia.

The specific objectives of the study are described in the following:

1. *Objective 1*: To test whether CaF in older adults are positively related with altered stress system activity, specifically overactivity of the sympathetic nervous system (SNS) and hypoactivity of the hypothalamus–pituitary–adrenal (HPA) axis, and higher levels of systemic low-grade inflammation. It will additionally be tested whether general psychological symptoms (stress, anxiety, depression) are similarly or more strongly associated with stress system activity.
2. *Objective 2*: To test whether a well-established CaF-reduction and exercise program reduce CaF will differentially affect stress system activity and reduce or slow down systemic low-grade inflammation. It will additionally be tested if and how general psychological symptoms (stress, anxiety, depression) are affected by the intervention.
3. *Objective 3*: To test for directionality of the brain-to-immune/immune-to-brain feedback system by investigating whether reduced CaF precede reductions in inflammation (or lower rate of increase) or whether reductions in inflammation precede reductions in CaF (or lower rate of increase) (or both). It will additionally be tested whether general psychological symptoms (stress, anxiety, depression) provide an alternative or additional pathway.
4. *Objective 4*: To test whether higher inflammation at baseline is predictive of lower success in the intervention group.
5. *Objective 5*: To test whether systemic and intracellular inflammation at T0 is associated with lower decreases of CaF from T0 to T1 and T0 to T2.

This project has been preregistered before the start of data evaluation; see osf.io/m84hq.

### Trial design {8}

**Fig. 1:**
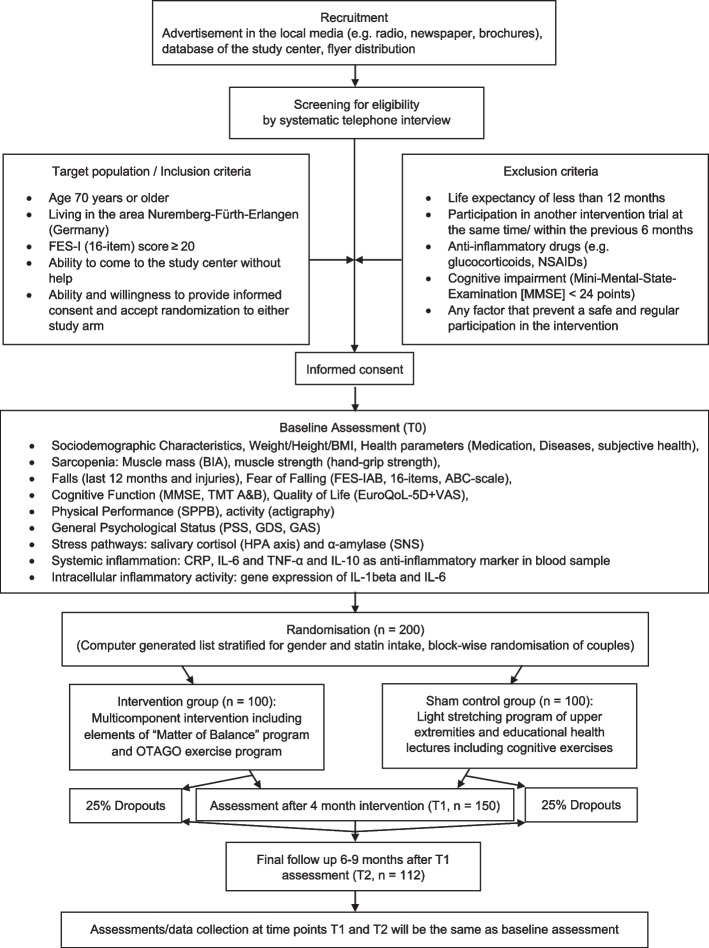
Flow diagram for FEARFALL

FEARFALL is a randomized controlled prospective trial with a duration of 3 years. Older adults will be randomly allocated into parallel groups of equal sizes (1:1). The study will be conducted double blind. Participants will be randomized into either intervention (IG) or sham control group (SCG), with the study designed to determine the superiority of the effectiveness of the IG compared to the SCG. While each employee of the project can perform recruitment and baseline assessment, the intervention will be carried out exclusively at the Institute for Biomedicine of Aging by trained exercise instructors. The Chair of Health Psychology will be responsible for the outcome assessment, allowing for blinding with regard to group allocation of the participants. All biological specimens will be analyzed by staff at Chair of Health Psychology in the laboratories physically separated from all other project locations. The study complies with the Declaration of Helsinki and Guidelines for Good Clinical Practice. The flow diagram for FEARFALL is shown in Fig. 1.

## Methods: participants, interventions and outcomes

### Study setting {9}

FEARFALL is a randomized controlled prospective study consisting of parallel groups. In this study, the investigators aim to enroll *n* = 200 community-dwelling older adults, aged 70 years and older, from the Nürnberg/Fürth/Erlangen region (Bavaria, Germany). Eligible participants must exhibit moderate to high levels of CaF, as defined by a score of at least 20 on the 16-item version of the Falls Efficacy Scale-International (FES-I) questionnaire, and possess adequate mobility to travel to the study center.

### Eligibility criteria {10}

FEARFALL’s eligibility criteria are shown in Table 1.

**Table 1:** Inclusion and exclusion criteria for FEARFALL study

Inclusion criteria:	Exclusion criteria:
• Age of 70 years or older • FES-I (16-item) score ≥ 20 • Ability to come to the study center without help • Ability and willingness to provide informed consent and accept randomization to either study arm	• Life expectancy of less than 12 months • Participation in another intervention trial (physical exercise or medical) at the same time or within the previous 6 months • Anti-inflammatory drugs (e.g., glucocorticoids, NSAIDs) • Cognitive impairment (Mini-Mental-Status-Examination [MMSE] < 24 points) • Any medical, psychiatric, or behavioral factors that in the judgment of the principal investigator may interfere with the study participation or the ability to follow the intervention

### Who will take informed consent? {26a}

All participants have to provide written consent after reviewing the participant information and engaging in a personal discussion with the trained study staff from IBA to address any remaining questions.

### Additional consent provisions for collection and use of participant data and biological specimens {26b}

For biological specimen, all participants have to sign the informed consent including data retention permission.

## Interventions

### Explanation for the choice of comparators {6b}

The SCG could be seen as standard care, because the participants will receive health lectures and light stretching exercises at the same frequency as the IG. All biological specimen will be collected and analyzed at the same time points in both groups.

### Intervention description {11a}

#### Study procedure

The general study design is that of a randomized controlled trial. After screening and providing written, informed consent, eligible participants will be randomized to participate in either the IG or the SCG of a 16-week intervention period (described in detail below). Randomization will be stratified by gender (female, male, or together as a couple), and statin intake, to ensure equal distribution between both groups. For each group, the intervention or control treatment will be followed by an 8-month follow-up period. This design allows to include 3 data collection time points: T0 at baseline, T1 after intervention or control treatment, T2 at 8 months post intervention. Post-intervention follow-up intervals are selected based on the anticipated trajectories of the development of psychological and psychobiological changes.

### Assessments

All measurements are collected at three time points, T0, T1, and T2, with the exception of demographic variables.

### Demographic variables

At the baseline assessment, general information including demographic information, age, gender, education level, income, current living circumstances, and family status will be collected.

### Biomedical, anthropometric and functional health variables

#### Health status

The health status will be recorded by questionnaire determining the number of diseases and medications.

#### Recent fall experiences

Whether participants have experienced fall events in the 12 months prior to enrollment will be assessed by a standardized questionnaire used in all our previous studies. If a fall has taken place, information about the severity of injuries will be obtained via questionnaire [33].

#### Sarcopenia

Muscle mass will be measured with bioelectrical impedance analysis (BIA; Seca Medical Body Composition Analyzer 515, Seca, Hamburg, Germany). Four-meter usual gait speed will be used as surrogate parameter for function and handgrip for muscle strength. Handgrip strength will be assessed with a Jamar hydraulic hand dynamometer (Sammons Preston Rolyan, Bolingbrook, IL, USA). All measurements will be done according to the EWGSOP2 recommendations [12].

#### Physical performance

Physical performance will be assessed via the Short Physical Performance Battery (SPPB), which is a brief performance battery based on a set of functional tasks related to the lower limbs [34]. Additionally, a one leg stand is performed for 10 s.

#### Changes of gait characteristics

In a subsample of participants, gait will be investigated with the GAITRite system (Rölke Pharma GmbH, Hamburg, Germany) as it also has very good psychometric properties demonstrated and is the gold standard for measuring gait characteristic [35].

#### Health-related quality of life (HRQoL)

HRQoL will be measured with the EURQOL (EQ-5D). The sum score and visual analogue scale (VAS, 0–100) will be determined [36] that is widely used in medical research.

#### Subjective health

Self-rated subjective health (SRH) will be obtained by a single question “In general, how would you rate your current health status?” with five response options: “very good,” “good,” “fair,” “bad,” or “very bad.”

#### Activity (actigraphy)

Objective Physical Activity (PA) will be assessed by actigraphy using the activPAL system (PAL technologies, Glasgow, UK). The activPAL system provides information on body postures e.g., sitting, lying, and walking. This information enables to obtain sedentary as well as physical activity behavior [37].

### Cognitive function

In order to test cognitive function and to ensure that all participants are able to comply with the study protocol and to provide informed consent to the study the Mini Mental State Examination (MMSE), as a part of the CERAD (Consortium to Establish a Registry for Alzheimer’s Disease), neurophysiological test battery will be applied. The MMSE is a widely used screening tool that provides a brief, objective measure of cognitive function and is one of the best validated tests of cognitive function among the elderly [38]. The MMSE cut-off score of < 24 will enable the participants to follow the CaF training, which is a cognitive-behavioral oriented training.

Furthermore, the Trail Making (Versions A and B) of the CERAD will be used to monitor executive function and cognitive flexibility [39].

### General psychological status

#### Depressive symptoms

In order to assess depressive symptoms, the Geriatric Depression Scale (GDS-15) will be used. GDS is appropriate for the age group in this project [40, 41].

#### Perceived stress

Perceived stress will be assessed with the general perceived stress scale (PSS). PSS is used for assessment of sub-chronic life stress in adults [42, 43] and is validated for older adults as well [44].

We will use the 10-item version as a global assessment of perceived stress, with which the sum scores could be assessed on a scale between 0 and 40. Higher sores indicate higher perceived stress with cutoff points 0–15 reflecting low and 15–40 high perceived stress for older adults aged 70 years or older [42].

Furthermore, perceived stress at saliva sampling time points will be rated on 5-point Likert-type scales. This self-reported questionnaire was previously used in research at the Chair of Health Psychology.

#### Anxiety

Anxiety will be operationalized with the Geriatric Anxiety Scale (GAS) [45]. GAS is a self-reported screening and assessment tool designed specifically for older adults. The GAS contains 25 scored items and 5 additional ones addressing specific areas of concerns often reported by older adults (for clinical application). Different components of anxiety, somatic, cognitive, and affective domain will be recorded in GAS scale. In this study, the German version of GAS (GAS-G) will be assessed [46]. Higher sum score indicates higher anxiety.

### Concerns about falling (CaF)

As a measure of CaF, the FES-I scale will be used, which is a 16-item questionnaire related to CaF during basic and more demanding daily activities among independent, community-dwelling older adults. The validated German version of the FES-I will be used [47]. In addition, to investigate the behavioral consequences of CaF the FES-IAB currently validated by Kruisbrink et al. [48], and to assess the perceived control of falling, the translated Updated Perceived Control over Falling Scale (UP-COF) scale will be used [49].

### Stress and stress-related pathophysiological processes

#### Stress system activity

Chronic stress has profound effects of the two main stress systems, HPA axis and SNS. We will assess basal activity of the HPA axis and SNS using non-invasive, at-home saliva samples measuring salivary cortisol for the HPA axis and salivary alpha-amylase (sAA) for the SNS, respectively, as for example documented in Rohleder et al. [50]. Based on our extensive experience with saliva sampling in home contexts [50–52], we will use the following procedure to ensure sufficient adherence of participants to the protocol. At first, an in-person explanation and practical instruction of the saliva sampling procedure will be conducted during the appointment. Second, a detailed set of written instructions will be provided along with a brief questionnaire asking participants for sampling times for reference at home. Participants will be asked to take three saliva samples at the following time points over two consecutive days: upon awakening, 30 min after awakening and at bedtime, for a total of six saliva samples. Participants are then asked to send all collected samples to the joint laboratory of the Chair of Health Psychology and the IBA using the pre-stamped envelope. Both, cortisol and sAA, are sufficiently stable at room temperature for this procedure [52]. Cortisol will be measured using a commercially available chemiluminescence immunoassay (Tecan, Hamburg), and sAA will be measured by an enzyme kinetic assay as described previously [50] using reagents by Roche Diagnostics (Mannheim).

#### Systemic inflammation

To assess systemic low-grade inflammation, we will take one blood sample at an appointment at the IBA using EDTA Vacutainers (BD, Heidelberg). As biomarker of stress-related pathophysiological processes, plasma concentrations of CRP, IL-6, and TNF-alpha will be assessed, and as a marker of anti-inflammatory activity, IL-10 will be measured with reagents by R&D Systems (Minneapolis, MN, USA).

#### Intracellular inflammatory activity

To obtain a more direct indication of the innate immune system’s contribution to systemic inflammation, gene expression of interleukin-1beta (IL-1beta), IL-6, IL-10, and I-kappa B will be analyzed. Therefore, RNA will be transcribed into cDNA, and gene expression analyses will then be performed according to standard procedures using real time quantitative RT-PCR as previously described, using primers and reagents from Life Technologies, or ThermoFisher [50, 53].

### Intervention

The intervention group will take part in a 16-week multicomponent exercise intervention.

During the initial 4 weeks, training sessions will take place twice a week, with supervised 60-min sessions at the IBA, where the participants will be familiarized with the components of the program. From week 5 to week 16, one supervised session per week will be provided at the IBA, and two 30-min sessions will be completed by participants as an unsupervised home program. An adherence rate of 75% in the intervention sessions will be considered as successful participation [54–57].

The multicomponent intervention will contain elements of gait, balance, and strength exercise to improve physical function and elements of the “Matter of Balance” program to reduce CaF [58]. The time points and frequency of the different exercise and behavioral components in the intervention are summarized in Table 2 and described in detail in the following.

**Table 2:** Time points and frequency of the intervention components

	**Month**				**Total**
**Intervention components**	1	2	3	4	
Strength training	2	2	1	1	6
Challenging balance training	2	2	2	2	8
Functional training		1			1
Gait training	1		1		2
Body awareness		1		1	2
CaF training		1	2	1	4
Home program	1			1	2

### Strength training

The strength training includes progressive strength exercises for upper and lower body and stretching exercises. The focus is on lower limb strengths and strength training will adhere to the effective Otago exercise program [59]. This program was repeatedly been shown to reduce falls in older adults. In short, the single exercise will include, e.g., wide leg squats knee extension, leg curl, and hip extension. All exercises will be conducted in two sets starting with five repetitions and increasing up to 10 repetitions. Participants will wear individually adjusted ankle weights based on the results of the self-rated exhaustion (Borg scale). As previously demonstrated, this sequence of exercises is successful in increasing strength in older adults [54].

### Challenging balance training

Challenging balance training will include static and dynamic balance exercise with progression, e.g., tandem stand and tandem walk. The increasing demands on balance control will involve reducing the amount of support. Initially, participants will hold onto a chair with both hands, then progress to using one hand, fingertips, and ultimately zero support. Additionally stable and unstable ground (e.g., foam mats) may be used as appropriate. The balance exercises will be tailored to each individual’s level.

### Gait training

Different gait components, e.g., step widths and step lengths, will be included in the gait training. The gait exercise will also include different rhythmical, spatial, and temporal components, as well as a dual task condition, e.g., walking and talking, and combination of gait and arm movements [54].

### Functional training

Functional training will include getting up from the floor by using the backward chaining methods [60].

### Body awareness exercise

Body awareness exercise will include body orientation practice. For instance, how can I attain an object without compromising my balance and disbalancing the positions of my center of mass and pressure? [54].

### CaF training

CaF training will include elements of the effective “Matter of Balance” training [58]. The “Matter of Balance” intervention is a cognitive-behavioral oriented training including goal setting elements for behavioral changes, e.g., to increase physical activity or integrate exercise elements of the home program into the individual daily routine. Misconceptions regarding the risk of falling, attitudes towards falls, thoughts, and CaF as well as negative and positive cognitive patterns, along with possible environmental hazards that could lead to falls will be examined. The elements include assessing daily situation with CaF with regard to thoughts, physiological symptoms, and behavioral aspects. Goal setting is to reduce CaF in these situations. In addition, self-efficacy regarding behavioral fall related aspects will be trained to reduce avoidance behavior in daily activities.

### Home program

Home program will be based on a brochure describing how to perform strength, balance, and gait exercises safely and regularly ([Online] Available from: https://www.gesund-aktiv-aelter-werden.de/service/mediathek/bewegung/sturzpraevention/ [Accessed 24 Nov 2023]) (https://www.gesund-aktiv-aelter-werden.de/service/mediathek/bewegung/sturzpraevention/). Materials from the “Bundeszentrale für gesundheitliche Aufklärung” could be regarded as usual care for fall prevention without exercise intervention. Participants will report the adherence to the home program in the intervention session, and further support will be elaborated for integrating the guidelines in the individual daily life.

### Sham control group

The sham control intervention will occur in the same frequency as the multi-component exercise intervention, over a 16-week period (as mentioned previously). Each session will contain a 40-min educational presentation and 20 min of light upper extremity stretching. In the SCG, the groups sizes will vary between 20 and 30 participants. As a fundamental aspect, the SCG will receive light stretching exercise and strength training of the upper extremities. Second part of the control intervention will be educational health lectures, as previously described before [61]. Topics of the educational and light stretching training sessions are detailed in Table 3.

**Table 3:** Topics of the control intervention

*Domain*	*Topic*
**Exercises**	
Light stretching of upper extremities	Standardized stretching of upper extremities with instruction of the home program
Strength training of the upper extremities	Exercises for strength training of upper extremities
**Health lectures**	
Demographic changes	Challenges and chances of an older population
Aging	Challenges and chances for older adults (healthy aging, living space adaptation, care order, living will, crime prevention, senior networks)
Medical topics	Polypharmacy, interpretation of laboratory test results, first aid, gait
Cognitive training	Training of short-term storage and retrieval, the training of memory strategies for long-term storage, attention and changes in aging
Nutrition	Challenges and chances of nutrition in older population

Participants will also receive a home-based light stretching booklet, brochures in health lecture topics, and a summary of the entire course.

### Criteria for discontinuing or modifying allocated interventions {11b}

Criteria for discontinuing the intervention for a participant is an adverse event such as a fall or accident resulting in severe injuries (e.g., fractures), which permanently prevents their participation in the intervention.

### Strategies to improve adherence to interventions {11c}

For saliva sampling, which will be undertaken by the participants themselves, personal meeting includes explanation and practical instruction of saliva sampling procedure. A detailed set of written instructions and a brief questionnaire asking participants for sampling times for reference at home are provided at this first appointment at IBA. If saliva samples were not returned after certain time, close communication via phone calls will be applied. Participants receive personal reminder calls for the assessments and testing appointments, if desired.

The participants will be introduced to each other during the intervention sessions to enable social bonding to the program. Special efforts will be made to keep the participants of the SCG in the project by handing out birthday cards and celebrating Christmas at a special event together.

### Relevant concomitant care permitted or prohibited during the trial {11d}

Measurement of salivary cortisol may be influenced by various factors, such as tooth brushing, eating, drinking caffeinated beverages, and regular physical activity. Therefore, participants are asked to refrain from consuming anything (except drinking) water, smoking, chewing gum, and brushing teeth for at least 1 h prior to saliva sampling. Excessive physical activity influences inflammatory biomarkers in the body. Therefore, participants are asked to avoid excessive physical activity 24 h before.

### Provisions for post-trial care {30}

Not applicable. All intervention components are validated and standardized procedures where no particular hazard is to be assumed. Additionally, an insurance is provided to all participants during the period of study participation.

### Outcomes {12}

As the primary outcome, the decrease in CaF after intervention (T1) measured by FES-I score in comparison to the SCG will be confirmed. All secondary outcome measures will be assessed at three time points (T0, T1, and T2) and changes between groups over time will be explored. Therefore, sarcopenia will be assessed by BIA, physical function will be tested by SPPB, and handgrip strength and physical activity will be tested by actigraphy. The VAS scale of EQ-5D will determine health-related quality of life. General psychological status will be explored with questionnaires for depression (GDS), perceived stress (PSS), and anxiety (GAS). Furthermore, MMSE and TMT A and B will determine cognitive function.

Pathophysiological parameters changes will be explored in blood samples such as levels of CRP, IL-6, TNF-alpha, and IL-10 for systemic inflammatory processes as well as gene expression of IL-1beta, IL-6, IL-10, and I-kappa B for intracellular inflammatory activity. In addition, stress system activity will be measured by changes in cortisol and alpha-amylase levels in salivary samples.

If a fall or accident resulting in severe injuries (e.g., fractures) occurs during the intervention period, it poses a potential risk to the participants and is therefore considered an adverse outcome.

### Participant timeline {13}

The participant timeline is shown in Table 4.

**Table 4:**
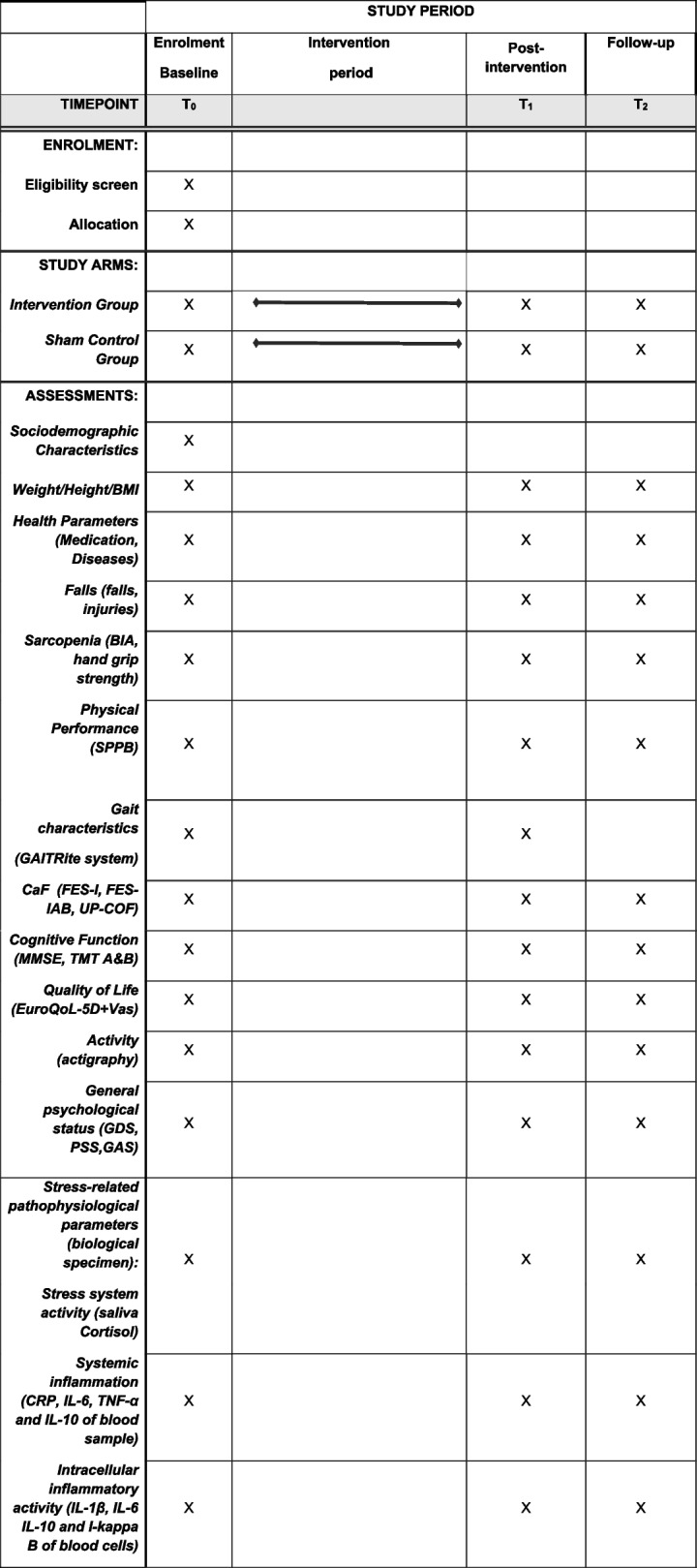
The participant time line for the schedule of enrolment, interventions and assessments

*MMSE* Mini Mental State Examination, *BIA* Bioelectrical impedance analysis, *SPPB* Short Physical Performance Battery, *CaF* Concerns about falling, *TMT* Trail Making Test, *VAS* Visual analogue scale, *FES-I* Falls Efficacy Scale-International Version, *FES-IAB* FES-I Avoidance Behavior, *UP-COF* Updated perceived control of falling, *GDS* Geriatric Depression Scale, *PSS* Perceived Stress Scale, *GAS* Geriatric Anxiety Scale, *CRP* C-reactive protein, *IL-6* Interleukin 6, *TNF-α* Tumor necrosis factor α, *IL-1β* Interleukin 1β

### Sample size {14}

Calculation of optimal sample size is based on two sets of available information. First, the required sample size for successful reduction of CaF was computed, and second, the required sample size for reduction of inflammatory markers in consequence of successful interventions was computed.

The authors are rather confident with regard to reduction of CaF: Based on previous intervention studies using similar procedures [54–56], a small-to-medium effect of around *f* = 0.15 is expected for reduction of CaF (indicated by FES-I). A priori power analyses using G-POWER (3.1.9.2.) assuming a statistical power of 0.95 and alpha error probability of 0.05 (assumed correlation among repeated measures of 0.50, and non-sphericity criterion ϵ set to 1). This indicates that *n* = 154 participants will need to be enrolled to achieve a successful reduction of CaF. Based on a drop-out rate of about 25–30% [56], *n* = 200 participants need to be randomly assigned to a group (i.e., *n* = 100 per group).

Second, if CaF are successfully reduced in the intervention group, it needs to be determined if the computed sample size of *n* = 100 per group is sufficient to detect the hypothesized cross-sectional and prospective associations of CaF with stress system activity and inflammation. As there are no published studies regarding this main prediction, the assumptions are estimated based on preliminary findings and expectations by the authors of what constitutes an acceptable effect size. Previous unpublished data for cross-sectional association of FES-I with IL-6 revealed that a sample size of *n* = 63 (assuming a statistical power of 0.95 and alpha error probability of 0.05) would be required to find a moderate-to-large effect (*r* = . 4); this also remains for multiple linear regression with up to six predictors (requiring *n* = 75). It can be concluded that the estimated sample size enrolling *n* = 200 participants with a drop-out of 25–30% will yield a sufficient final participant number to not only successfully reduce CaF but also to find the hypothesized associations using bivariate correlation and multiple linear regression analyses. Support of this finding comes from a study showing that frequent aerobic exercise training had a medium-to-large effect with regard to reducing inflammatory mediators [62]. It should be noted that this study employed a more frequent (three times/week) and longer intervention (10 months) than planned in our study; therefore, we do expect a smaller effect.

### Recruitment {15}

Participants will be recruited by established strategies via advertisement in local media (e.g., newspaper, brochures) and using an already existing data pool of older adults from other studies. This recruitment strategy has been successful in the past in other projects. In addition, flyers are displayed in public local places (shops, library and physicians) as well as advertisements in public transportation.

## Assignment of interventions: allocation

### Sequence generation {16a}

Randomization will be stratified by gender, and statin intake, to ensure equal distribution between both groups, while couples will be block-wise randomized to ensure participation together in one group. The randomization lists with block sizes 4, 6, and 8 were computer-generated via an online tool (Sealed Envelope Ltd. 2021. Create a blocked randomization list ([Online] Available from: https://www.sealedenvelope.com/simple-randomiser/v1/lists [Accessed 12 Aug 2022] [63] by study personnel who was otherwise not involved in the planning of the study design).

### Concealment mechanism {16b}

The randomizing person receives only the stratification information to assign the intervention. The allocation sequence is hidden in a read-only, password-protected spreadsheet. The revelation of a new group allocation is automatically documented by the system including username, date, and time. Exposed group allocations cannot be altered or erased. The recruiting team has no access to the randomization list.

### Implementation {16c}

After signing the written, informed consent and completing baseline assessments during the first in-person visit conducted by trained study staff, randomization is carried out by enrolling participants in concealed randomization lists by a separate member of the intervention team who only receives the necessary information for stratification.

## Assignment of interventions: blinding

### Who will be blinded {17a}

The study will be conducted as a double-blind study. Participants will be randomized into either the IG or the SCG with the information that the aim of the study is to compare two different intervention methods. While each employee of the project can perform recruitment and baseline assessment, the personnel at the IBA will solely conduct the intervention. The Department of Health Psychology will be responsible for the outcome assessment, allowing for blinding with regard to group allocation of the participants. All biological specimens are labeled by a participant code only, which allows blinding during laboratory analysis.

### Procedure for unblinding if needed {17b}

We do not anticipate any requirement for unblinding, but, if required, the principal investigator will have access to group allocations, and any unblinding will be reported.

## Data collection and management

### Plans for assessment and collection of outcomes {18a}

Data collection procedure is shown in Table 4. Except of sociodemographic characteristics and cognitive function, all questionnaires and laboratory and functional tests are performed in-person at each visit, respectively. Data collection will be finished in the 2nd quarter of 2025.

After completing data collection for each time point, data entry will be performed contemporaneously to ensure identification of missing data and any discrepancies. This enables to record missing data or incorrect inputs from participants. Prior to hypothesis testing, all data will be cleaned, inspected for outliers, and tested for normality of distribution. Composite scores will be computed were appropriate, e.g., for questionnaires. Laboratory data will be quality checked using standard procedures, including intra- and inter-assay variability. All biomarkers will be analyzed in duplicates, as is done in standard procedures in the laboratory. Laboratory values not complying with quality control requirements will be repeated. Means and standard deviations for all variables will be computed, and preliminary analyses testing for age and gender effects, pre-intervention group differences, and time effects of repeated samples will be performed.

### Plans to promote participant retention and complete follow-up {18b}

Each participant receives their own data at each in-person visit as well as a summary of all their data at the end of the study. Reminder calls and appointment invitations are sent to promote participant completion of the intervention and follow-up assessments.

### Data management {19}

With regard to data management, we will follow the current research data policy of Friedrich-Alexander-Universität Erlangen-Nürnberg (released in December 2019). All data will be handled in compliance with current German and European data protection laws and will be subject to our standardized data safety and monitoring plan. Ethics approval has been obtained from the local IRB (“Ethikkommission der Medizinischen Fakultät der FAU”), #317_20B, August 26, 2020, and all data collection, storage, and the local data protection authority (“Datenschutzbeauftragter der FAU”) will evaluate protection procedures. The study protocol complies with the Declaration of Helsinki and Guidelines for Good Clinical Practice. This study follows the FAIR principles (“Findable, Accessible, Interoperable und Re-Usable”) for data management. All data will be pseudonymized, i.e., a numerical code will be generated and used as a data identifier. Personal information will be stored in data file separate from all other data, and only the principal investigators will have access to identifying personal information. Participants will be granted the right to ask for their data to be deleted as long as the data is not completely anonymized. One year after completion of data analyses, all personal information will be destroyed. De-identified data will be stored on secure file servers for 10 years according to DFG recommendations. De-identified data will be made available through a public repository to be defined (e.g., ZPID). Data monitoring protocols will be established to ensure that any adverse events are reported by study coordinators and PhD students to the responsible PI (Drs. Britting and Freiberger for the intervention part, Prof. Dr. Rohleder for the laboratory part). Responsible PIs will notify the relevant supervising bodies (IRB; data protection authority) within 1 week. All PIs will establish a monthly data safety meeting. Data entry will be double-checked by a trained study nurse and staff of the IBA to guarantee correctness, completeness and plausibility of the provided information. The record of the results in the electronic database and its countercheck should be performed within 1 month of the elicitation to enable a re-collection of missing or wrong inputs from the participants. After completion, the anonymized electronic database will be made available for other researchers upon request. It will be checked if the database could also be saved in a public repository (e.g., DRYAD https://datadryad.org/).

### Confidentiality {27}

Separately from all other data, personal information will be stored in a password-protected data file.

### Plans for collection, laboratory evaluation and storage of biological specimens for genetic or molecular analysis in this trial/future use {33}

Saliva and blood samples will be collected and stored at specific temperatures for analysis. Saliva samples will be taken at IBA and kept at – 20 °C, while blood samples will be stored at − 80 °C after subsequent processing. Laboratory analysis will take place at shared facilities between the IBA and the Chair of Health Psychology following the completion of sample collection. All biological samples will be preserved for potential re-analysis until the end of the study.

## Statistical methods

### Statistical methods for primary and secondary outcomes {20a}

IBM SPSS® Statistics for Windows, Version 29 software (IBM Corp., Armonk, NY, U.S.) will be used for all statistical analyses. Descriptive statistical analysis will be performed for participants’ characteristics as mean ± standard deviation or median and interquartile difference for continuous variables. Absolute numbers and percentages will be presented for dichotomous and categorical variables. Prior to data analyses, data will be checked for outliers and normality. For the evaluation of primary outcome, the reduction of CaF at T1 will be investigated by FES-I Score in comparison to CG.

All hypotheses of this study are described in detail in the “Objectives {7}” section and are preregistered at https://osf.io/m84hq.

### Interim analyses {21b}

Not applicable. No interim analysis is planned due to the use of validated and standardized procedures.

### Methods for additional analyses (e.g., subgroup analyses) {20b}

Not applicable. No subgroup analysis is planned.

### Methods in analysis to handle protocol non-adherence and any statistical methods to handle missing data {20c}

Statistical analysis will be conducted with participants who adhere to the intervention, while non-adherent participants will be excluded from the analysis. Adherence to the intervention will be monitored at each session. Participants will be considered “adherent” to the intervention if they attend at least 15 sessions (75%). A drop-out rate of 25% was considered in the power calculation; therefore, per-protocol analysis will be conducted.

### Plans to give access to the full protocol, participant-level data and statistical code {31c}

After the study results are published, participant’s data and statistical code will be accessible upon reasonable request. We will also examine the possibility of storing the database in a public repository (e.g., DRYAD https://datadryad.org/).

## Oversight and monitoring

### Composition of the coordinating center and trial steering committee {5d}

Not applicable. In this study, no formal committees are set up. Responsibility and organization are shared on the principal investigators and are coordinated in weekly meetings.

### Composition of the data monitoring committee, its role and reporting structure {21a}

Not applicable. In this study, no formal committees are set up. Responsibility and organization are shared on the principal investigators and are coordinated in weekly meetings.

### Adverse event reporting and harms {22}

Adverse events, e.g., falls and injuries during training, will be recorded.

### Frequency and plans for auditing trial conduct {23}

Not applicable. In this study, no external audit will be conducted as we are not subject to any drug trial regulations. Random data checks will be performed by the principal investigators who are not involved in data collection.

### Plans for communicating important protocol amendments to relevant parties (e.g., trial participants, ethical committees) {25}

The study protocol was approved by the ethics committee vote (number 317_20B) of the Friedrich-Alexander-Universität Erlangen-Nürnberg and complies to the Declaration of Helsinki and Guidelines for Good Clinical Practice. This study was registered at German Clinical Trials Register (identifier: DRKS00029171). Protocol amendments are communicated to both the Ethics Committee and the German Clinical Trials Register in writing.

### Dissemination plans {31a}

The dissemination plan includes publication of the results in national and international journals as well as dissemination in conference contributions.

## Discussion

Maintenance of physical function, mobility and ability to live independently are important goals for older adults. However, this is counteracted by age-related loss of muscle mass, strength, and function. Furthermore, this degenerative process can be reinforced if the older person avoids physical activity and exercise due to fear of falling. In this study, it will be established that CaF are not only leading to decreased physical activity but are also associated with elevated levels of inflammation. Consistent with that, long-term exposure to adverse psychological conditions, including chronic stress and anxiety, has been linked to the upregulation of inflammatory activity. This chronic low-grade activation of the immune system was shown to intensify the decay of skeletal muscle. Therefore, a vicious, feed-forward cycle of fear of falling, inflammation, loss of muscle mass, and decreasing physical function is created that ultimately results in negative health outcomes, i.e., falls, dependence and death. To the best of the author’s knowledge, no published study to date has directly tested whether CaF are related to stress system activity or reactivity or to any marker of inflammation.

## Potential risks and limitations

Many factors are known to potentially influence saliva cortisol measurement, such as tooth brushing, eating, drinking caffeinated drinks, and ordinary physical activity. Therefore, participants should refrain from consuming anything (except drinking) water, smoking, chewing gum, and brushing teeth for at least 1 h prior to saliva sampling. The time points for saliva sampling are specified and explained in person. Participants might not always adhere to these requests, which might influence the results.

In this sample is a high proportion of comorbidity, which might influence concentrations of biomarkers in blood and saliva samples. Polymedication and intake of, e.g., beta blockers interfere with the function of the autonomous nervous system [64].

A large number of factors may influence PSS as well as cortisol concentration: age, sex, smoking, BMI, ever diagnosis of depression, ever diagnosed cardiovascular disease, time of day.

## Clinical and practical implications

In this study, CaF and stress system (re-)activity or inflammatory markers will be assessed in the context of a multi-component intervention in community-dwelling older adults. This allows to better understand underlying pathophysiological mechanisms in the body. Understanding the role and directionality of the effects of inflammation on CaF would significantly increase our knowledge of the underlying causes of age-related loss of mobility. In addition, this opens up a number of avenues that would increase the efficacy of interventions aimed at increasing mobility and functional health in later life. The gained knowledge will help to improve interventions for this target group.

## Conclusion

This study will be the first to test whether CaF are related to stress system activity or reactivity or to any marker of inflammation in the context of a multi-component intervention with exercise training and cognitive-behavioral components addressing CaF. The reduction of specific CaF or general psychological symptoms will reverse alterations in stress systems and/or slow down low-grade inflammation. Changes in activity, as well as psychological and biological pathways leading from CaF to muscle loss, will be measured to disentangle the individual contribution to sarcopenia and to provide an additional pathway to break or slow-down the vicious cycle of CaF and sarcopenia.

## Trial status

FEARFALL is at the recruitment stage when this manuscript is completed. Recruitment starts in September 2022 and will be completed in May 2024. This study protocol is version 1.0, dated 13 March 2024. The end of this trial is expected in August 2025.

## Supplementary Information

Supplementary Material 1.

## Data Availability

The final data set is available for the principal investigators. After study completion, data access can be granted upon request. It is planned to make the database accessible in a public repository (e.g., DRYAD https://datadryad.org/).

## Abbreviations

BIA: Bioelectrical impedance analysis
CaF: Concerns about falling
CAR: Cortisol awakening response
CRP: C-reactive protein
FES-I: Falls Efficacy Scale International
FES-IAB: FES-I Avoidance Behavior
FoF: Fear of falling
GAS: Geriatric Anxiety Scale
GDS: Geriatric Depression Scale
HPA: Hypothalamus-pituitary-adrenal
IBA: Institute for Biomedicine of Aging
IG: Intervention group
IL: Interleukin
MCI: Mild cognitive impairment
MMSE: Mini Mental State Examination
PSS: Perceived Stress Scale
sAA: Salivary alpha-amylase
SCG: Sham control group
SNS: Sympathetic nervous system
SPPB: Short Physical Performance Battery
TMT: Trail Making Test
TNF-alpha: Tumor necrosis factor alpha
UP-COF: Updated Perceived Control over Falling Scale
VAS: Visual analogue scale
